# Myeloid regulatory cells: cross talk of innate and adaptive immunity to maintain the intestinal immune homeostasis in inflammatory bowel disease

**DOI:** 10.1186/2194-7791-2-S1-A4

**Published:** 2015-07-01

**Authors:** Jan Däbritz, Toni Weinhage, Georg Varga, Timo Wirth, Jan M Ehrchen, Louise M Judd, Trevelyan R Menheniott, Andrew S Giraud, Dirk Foell

**Affiliations:** University Children's Hospital Münster, Münster, NRW, Germany; Murdoch Children's Research Institute, Melbourne, VIC Australia

## Meeting abstract

Intestinal monocytes/macrophages sustain the intestinal immune homeostasis and might be an attractive therapeutic target for the management of inflammatory bowel disease (IBD). Granulocyte macrophage colony-stimulating factor (GM-CSF) exerts beneficial effects in intestinal inflammation and promotes STAT3-mediated expansion of myeloid-derived suppressor cells (MDSCs). We explored whether GM-CSF mediates its beneficial effects in IBD via myeloid regulatory cells (Mreg).

Here we show that GM-CSF i) provokes non-classical monocyte activation; ii) drives monocytes towards an anti-inflammatory phenotype; iii) enhances innate immune functions; iv) primes monocyte responses to secondary microbial stimuli; and v) accelerates epithelial healing via monocytes. GM-CSF-activated monocytes (GMaM) show therapeutic activity in T cell-induced colitis in *Rag1*^*-/-*^ mice with increased production of IL-4, IL-10, IL-13 and decreased production of IFNγ in LPMCs. Confirming this finding, GMaM attract T cells and shape their differentiation towards Th2 cells *in vitro*. In addition, GMaM induce regulatory (Foxp3^+^) T cells (Treg) *in vitro* and adoptive transfer of GMaM in chronic DSS-induced colitis ameliorates disease *in vivo* with accelerated gut homing of GMaM and induction of colonic Treg. Myeloid-cell specific STAT3 activation protects gp130^757F/F^ mice from colitis via MDSC expansion and increased production of suppressive and protective cytokines. LysMcre/STAT3^flox^ mice with myeloid-specific STAT3-deficiency show opposite effects and are not protected from colitis. Additionally, MDSCs of gp130^757F/F^ mice produce significantly more IL-4, IL-10 and IL-13.

In summary, beneficial effects of GM-CSF in IBD may possibly be mediated through reprogramming of monocytes and MDSCs via enhanced innate immune functions as well as regulation of adaptive immunity. Our findings support the exploration of stimulating rather than suppressive therapies for patients with IBD and underpin that Mreg might become a promising novel cell-based therapeutic option.

